# The Relationship Between Built Environment and Mental Health of Older Adults: Mediating Effects of Perceptions of Community Cohesion and Community Safety and the Moderating Effect of Income

**DOI:** 10.3389/fpubh.2022.881169

**Published:** 2022-06-17

**Authors:** Rongrong Zhang, Xiong He, Ying Liu, Ming Li, Chunshan Zhou

**Affiliations:** ^1^School of Geography and Planning, Sun Yat-sen University, Guangzhou, Guangzhou; ^2^Land Consolidation and Rehabilitation Center MNR, Beijing, China

**Keywords:** built environment, older adults, mental health, perception of community cohesion, perception of community safety, income

## Abstract

Many studies revealed a significant correlation between low-density built environment and the mental health of older adults in developed countries. However, scholars and decision-makers recently began to pay close attention to the effect of this relationship in high-density built environments and in developing countries. Using point-of-interest (POI) data from Baidu and data on 20 communities in Guangzhou, China, which were collected through a questionnaire survey, this study aimed to examine the relationship between built environment and the mental health of older adults as well as the physiological–psychological mediating paths between the two, so as to enrich the research on population aging in the high-density urban context in developing countries. The findings indicated that facility accessibility and distance to parks significantly positively correlated with the mental health of older adults and the number of public transit stations, and the distance to these stations significantly negatively correlated with the mental health of older adults. Also, the perceptions of community cohesion and community safety had a significant mediating effect between the built environment and the mental health of older adults. Furthermore, the moderating effect analysis results verified the moderating effect of income: with an increase in income, the perception of community cohesion enhanced the protection of the mental health of older adults and reduced the mediating effect of the perception of community safety. The results provided a reference for policy-makers and urban planners in their efforts to plan and build health-supporting communities and a healthy aging society.

## Introduction

Population aging is one of the major challenges facing most countries worldwide (1), especially China (2). According to data from the Seventh National Population Census of China conducted in 2020, 264 million are 60 years and older, accounting for 18.7% of the national population and representing an increase of 5.44 percentage points from 2010, when the Sixth National Population Census was completed (seventh national population census). It is predicted that, by 2050, the number of older adults in China will peak at 487 million and account for 34.9% of the total population. China will face tremendous pressure brought about by a large aging population. With the rapid growth of the aging population, the Chinese government has proposed to build a society of “healthy aging,” and protecting the physical and mental health of older adults is a key component of this initiative (3). In reality, however, the mental health of older adults does not present an optimistic outlook. Mental health usually refers to a state of happiness in whichindividuals are aware that they can cope with normal stresses of everyday life, perform work effectively, and contribute to their community (4). According to the National Mental Health Development Report of China (2019-2020), nearly one third of the older adults in China have psychological disorders urgently needing interventions (5). The mental health of older adults has become a topic worthy of deep concern.

Older adults are more likely to experience health-related changes and challenges as age advances, making them potentially more sensitive and susceptible to the residential environment than other age groups (6–8). Experts and scholars from various fields, such as planning, medicine, sociology, and geography, have begun to consider the importance of the community environment with regard to the mental health of older adults (9–12). A growing body of evidence indicates that the community environment may impact the mental health of older adults (13–16). For example, a study on New York residents has indicated that people who live in a lower-quality built environment are more likely to suffer from depression (17). Therefore, building or developing built environment in communities has been gradually incorporated into more public health projects as an important measure to improve the mental health of residents (2).

However, almost all existing research or projects have been conducted in developed countries (1, 11, 17), and limited research evidence pertains to developing countries, such as China. On the one hand, developing countries are also facing the problem of aging (18). On the other hand, the built environment in developing countries is very different from that in developed countries. First, as developing countries are still in the process of urbanization, many people gather in cities for a long time, resulting in a very high population density (19). Second, compared with auto-dependent cities in the West, public transport is the main travel mode of urban residents in most developing countries such as China (20). Moreover, the distance between the daily activities of residents and destinations is still very long, although the development is extensive (21).

Therefore, research data from non-developed countries and low- and medium-income countries may reveal situations that cannot be found in developed countries and offer new insights into the mechanism driving these phenomena. Therefore, Guangzhou, a megacity in China, was chosen for a case study. This study used data collected from a questionnaire survey to investigate how the community environment affected the mental health of older adults in China. This study provided a reference for enriching related research in the context of Chinese cities and for active spatial intervention and cultivation of a healthy aging society.

### Background

The built environment refers to the objective material environment built by human beings for daily living, work, and entertainment (22). It mainly includes building units (e.g., houses, schools, and workplaces), open spaces (e.g., parks, squares, and recreational venues), infrastructure (e.g., transportation systems), and public service facilities (shopping malls, stadiums, and libraries) (23). Most researches on the relationship between built environment and mental health focus on the built environment at the community level (24), that is, the level closest to residents (25) in the ecosystem theory, as the built environment at this level encompasses direct interactions with residents (26). Also, abundant literature exists on the effect of the built environment of the community on mental health. Putrik, de Vries (27) found that residents living close to the railway were exposed to a high number of stressors and reported worse mental health. Remes, Lafortune (28) explored the relationship between residential environment and major depressive disorder, and found that women living in deprived areas were more prone to anxiety, while men living in an disadvantageous environment are more likely to have depression. However, a study of young adults conducted in the UK demonstrated that greater loneliness was associated with perceptions of lower collective efficacy and greater neighborhood disorder but not with more objective measures of neighborhood characteristics (29). Studies have also shown that the mental health of older adults is particularly associated with their residential environment, and the relationship between them is still not fully understood (11). A study conducted by Maas, Spreeuwenberg (30) in the Netherlands indicated that compared with older adults living in communities with less green space, those living in areas with more green space were less likely to suffer from depression. Similarly, Zhou, Yuan (31) investigated the linkage between greenness and the well-being of older adults, and found that community greenness was positively correlated to regular social interactions among older adults and hence positively linked to their mental health. Some studies, however, questioned the positive effect of green spaces because of the risk associated with them. These studies argued that green spaces might provide a hideout for criminals, increase crime, and cause stress to nearby residents, which were detrimental to mental health (32). Some studies verified the positive impacts of residence density, street connectivity, housing, and community quality on mental health (33, 34), while others proposed that the impact was negative (35). Furthermore, some studies argued that these relationships were non-linear and that more empirical research was needed to clarify the relations (36).

The built environment may not only be directly related to people's mental state but also affect residents' perception of the environment, thus explaining mental health outcomes to a certain extent (37). The physiological–psychological path is one of the major mechanisms through which the sense of unsafety and disorder increases chronic physical stress on people, thereby damaging mental health. The perception of unsafety is also considered an important source of stress (38, 39). Perceptions of unsafety and high degrees of chaos in the surrounding environment generate feelings of helplessness and fear and directly or indirectly increase the perceptions of suffering and mental stress (40, 41). For example, the lack of public spaces in a community deters residents from interacting with others and engaging in social activities, leading to a higher degree of distrust and fear toward the community (37, 42, 43). Wang, Yuan (44) found that perception of community safety benefited residents' mental health by offering residents a favorable place to interact with their neighbors and participate in neighborhood activities. Robinette, Charles (45) found that people who perceived lower community safety had more health problems 10 years later than those perceiving more community safety. Furthermore, community cohesion is another connection between a built environment and mental health. The built environment of a community can, in some context, provide a free, relaxed space for social interactions, enhance social cohesion within a community, protect the mental health of residents, and hence ease stress. For example, a better perception of community cohesion enhances mutual trust and unity among neighbors (46) and improves mental health and well-being by promoting social and physical activities and buffering against the negative effects of stress (47–49). Therefore, the perception of community cohesion plays an important role as a buffer between a community environment and mental health, that is, it can offset some of the negative effects of an adverse community environment on mental health (e.g., depression) (12, 50). Some researchers attempted to incorporate the perceptions of community cohesion and community safety into models to investigate the relationship between built environment and mental health and found that the perceptions of community cohesion and safety might play a mediating role in the relationship between a built environment and mental health. Existing research, however, merely incorporates the two into the same model. The mediating role of the perception of community cohesion or the perception of community safety as an independent factor has not been established (24).

In addition, previous studies on built environment and the mental health of older adults have been conducted in developed countries in a low-density urban context (1, 11, 17, 51). As a developing country, China has an urban context distinct from that in Western countries, as evidenced by the high population density and high building and transportation network density in China (18, 19, 52). As one of the megacities of China, Guangzhou is representative of the high-density urban context. Its permanent population has been increasing substantially, and the population is rapidly aging (53). Therefore, the impact of the built environment in Guangzhou on older adults may differ from that seen in developed countries that have low population densities. Choosing Guangzhou for this empirical study might help guide countries with high population densities in their strategic planning for building livable cities that help improve the mental health of older adults. This case study might also help optimize public policies that focus on developing a healthy aging society.

Income is prominent, in addition to the environment, among the multiple factors that may affect the mental health of older adults (3). Irrespective of the social environment in China or in the Western countries, older adults with a lower socioeconomic status are more susceptible to the pressure brought about by the environment, and their state of mental health is usually inferior (54). For example, a study conducted by Maas, Verheij (51) in the Netherlands indicated that the relationship between green spaces and anxiety was the strongest among older adults with a lower socioeconomic status. In investigating the impact of the perception of a built environment on depression among older adults in China, Pan, Liu (3) found that monthly income had a significant moderating effect on the significant negative correlation between perceptions of the built environment and depression, in other words, the impact of the perceived built environment on depression weakened among older adults with a higher monthly income. Therefore, we hypothesized that the relationship between perceptions of the built environment (i.e., perceptions of community cohesion and community safety) and the mental health of older adults was moderated by income.

**Figure 1:**
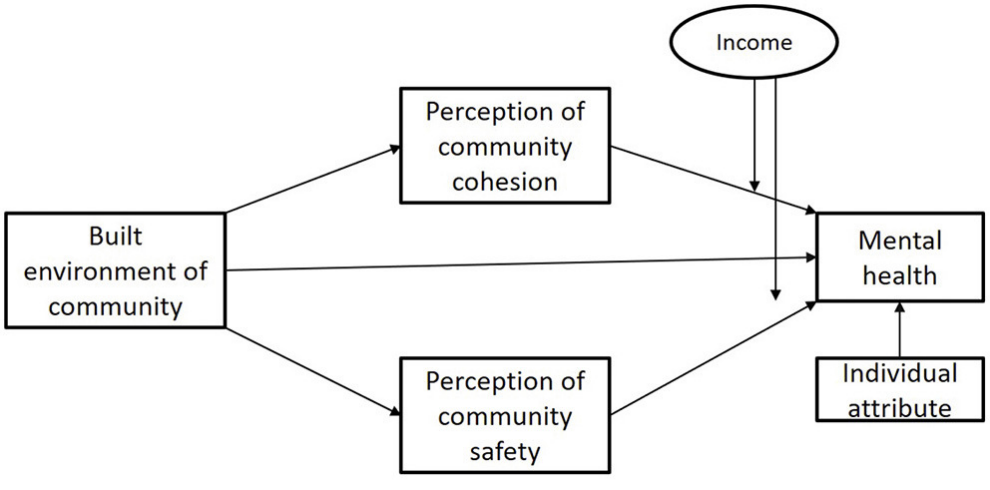
Research framework.

We developed a research framework (Figure 1) based on the environment stress theory to guide the investigation of how the built environment affected mental health through the perception of community cohesion and perception of community safety so as to fill the gap in existing research and enrich research on aging. More specifically, this study aimed to answer the following questions: (1) What is the relationship between a built environment and the mental health of older adults? (2) Does the perception of community cohesion or the perception of community safety have a mediating effect? Is there a difference between the impacts caused by these mediating effects? (3) Are the relationships between the perceptions of community cohesion and community safety and the mental health of older adults moderated by income level?

## Data and Methods

### Data

**Figure 2:**
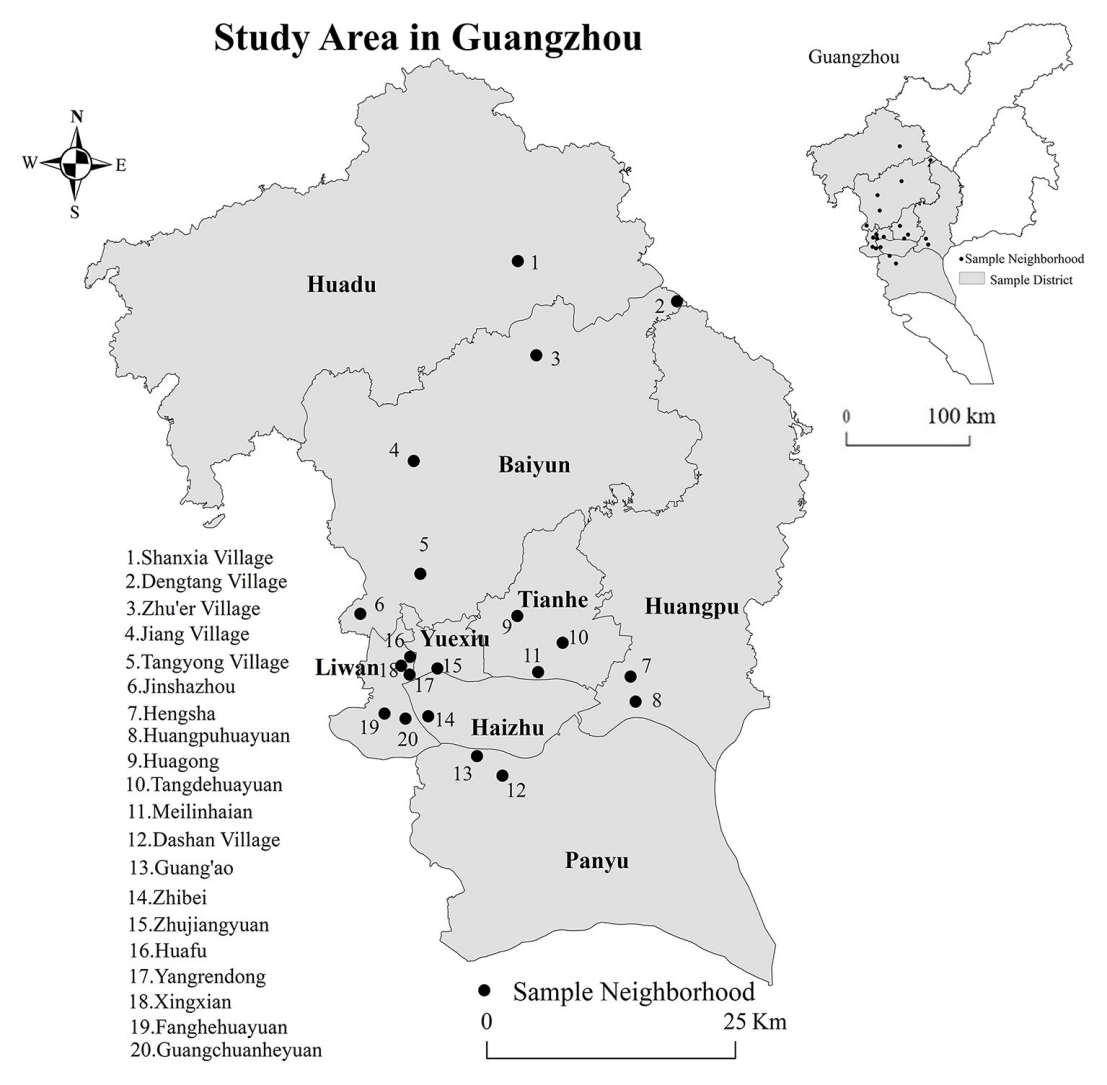
Location of surveyed communities. Map from the National Natural Resources and Geospatial Basic Information Database (https://www.sgic.net.cn/web/geo/index.html#/Home).

By the end of 2019, 1.7551 million people were aged 60 and above in Guangzhou, accounting for 11.47% of the total population. From December 2018 to April 2019, we conducted a questionnaire survey of older adults aged more than 60 years living in Guangzhou for more than 6 months. A multistage stratified probability proportionate to population size sampling technique (PPS), which enabled each unit to have the probability of being selected in proportion to its size (55), was adopted toselect respondents. First, Guangzhou was divided into six types of social aging areas (Table 1) on the basis of previous findings (56), including concentrated distribution areas of older adults in old neighborhoods; in government agencies, enterprises, and institutions;in urban villages; and in new development areas of a younger generation, scattered distribution areas of retired older adults in education and scientific research units, and mixed population distribution areas. Subsequently, the factor analysis was carried out on the selected 71 variables when defining six types of social aging areas. Five main factors with a cumulative variance contribution rate of 81.04% were extracted in our previous study (56). Thus, 18 subdistricts with the highest scores of five main factors among these six social aging areas were selected. Next, 20 communities with more than 10% of older adults within these 18 subdistricts were selected, covering six housing types: institutional, affordable, historic, rural self-built, commercial, and urban village housing (Table 1; Figure 2). Second, the number of questionnaire in each community was based on the proportion of older adults. The respondents from each sample community were randomly selected.

**Table 1:** Surveyed communities.

**Social area type**	**Definition**	**District**	**Subdistrict**	**Community**	**House type**	**Number of questionnaire completed**
High-concentration area of older adults in an old urban area	The older adults retired from ordinary work units are concentrated; the characteristics of elderly families are significant	Liwan	Hualin	Xingxian	Historical	25
			Longjin	Huafu	Historical	10
			Lingnan	Yangrendong	Historical	28
		Yuexiu	Zhuguang	Zhujiangyuan	Historical	68
Gathering areas for older adults who have retired from government enterprises and institutions	The older adults retired from government enterprises and institutions are concentrated	Liwan	Baihedong	Guangchuanheyuan	Danwei	108
		Haizhu	Nanshitou	Zhibei	Danwei	126
		Huangpu	Huangpu	Huangpu	Commercial housing	29
		Tianhe	Yuancun	Meilinhaian	Commercial housing	36
Scattered distribution area of older adults who have retired from educational and scientific research institutions	A cluster of educational and scientific research institutions, where older adults retired from these units are scattered	Tianhe	Wushan	Huagong	Danwei	87
Mixed population distribution area	The population of each occupation is roughly equally distributed, and the older adults are mainly retired, also including some rural older adults	Liwan	Dongjiao	Fanghehuayuan	Affordable housing	22
		Baiyun	Jinsha	Jinshazhou	Affordable housing	90
		Panyu	Luopu	Guangao	Commercial housing	17
		Huangpu	Dasha	Hengsha	Commercial housing	29
Concentrated distribution area of rural older adult population	The older adults in rural areas, whose main source of livelihood is labor income and family support, are concentrated in households with more than three generations living under the same roof	Baiyun	Zhongluotan	Dengtang	Rural village	52
		Baiyun	Zhuyuan	Zhuer	Rural village	32
		Baiyun	Jianggao	Jaingcun	Rural village	19
		Huadu	Huadong	Shanxia	Rural village	47
New development zone with young population	Young migrant workers engaged in production, transportation, and service industries are concentrated, while the number of older adults is small	Baiyun	Xinshi	Tangyong	Urban village	44
		Panyu	Dashi	Dashan	Urban village	55
		Tianhe	Tangxia	Tangdehuayuan	Affordable housing	8

After the sample communities were determined, we submitted this survey activity and questionnaire to the Institutional Review Board of the School of Geography and Planning, Sun Yat-sen University, for review and approval. Then, we got in touch with the neighborhood committees of the sample communities. The neighborhood committees agreed to let us enter the community after being informed that all the survey data were used for academic research. Next, we conducted face-to-face interviews with each older adult for about 30 min in public places in the community. All respondents involved in this study gave their informed consent and described their feelings and thoughts in the past 4 weeks. They were asked to assess the importance of the built environment in their communities and their satisfaction with each environmental element. The respondents were also asked to evaluate statements about interpersonal relationships and safety in their communities. At the same time, they were also asked to provide some personal and demographic information about themselves and their families, such as income, housing, marital status, and other information. We finally enrolled 1000 study participants and a total of 932 valid questionnaires were completed (Table 1); the completion rate was 93.2%.

### Variables

#### Mental Health

Mental health is defined as a state of emotional well-being. It was measured using mental health parameters in the 36-item Short-Form Health Survey (SF-36) (57). It included eight questions to assess the mental health–related feelings of respondents over the past 4 weeks: “Your mind has been in a healthy state,” “You have felt calm,” “You have felt happy,” “You have been able to concentrate on your work,” “You haven't felt stressed,” “You haven't felt nervous,” “You haven't felt dejected,” and “You haven't felt energetic” (Appendix A). Based on the Likert scale, each question had five options from 1 to 5: strongly disagree, disagree, general, agree, and strongly agree. Considering that the explained variable was basically normally distributed (58) according to previous studies (31, 58, 59), it could be regarded as a continuous variable. Therefore, the total score of mental health was obtained by adding up the scores of each question, ranging from 8 (worst health outcome) to 40 (best health outcome). Cronbach's alpha in the mental health project in this study had good internal consistency (0.923).

#### Built Environment

The older adults spent more time in the community after retirement and were the most affected by the built environment around the community (60). At the same time, considering the average walking speed of the older adults, the built environment of the community selected in this study was a 1-km buffer zone defined by the location of the community committee (53). In the existing studies, the measurement of the built environment was mostly based on the “5D” proposed by Ewing and Cervero (61), that is, density, diversity, design, accessibility, and distance to destination. Considering the previous findings (22, 53, 62) and the availability of data, we selected “4D” (density, diversity, accessibility, and distance to destination) from “5D” to measure the built environment of a community in this study (Table 2), which could be obtained from the seventh national population census in China and POI data from Baidu.

**Table 2:** Built environment of the community.

**Type**	**Name of variables**	**Definition**
Density	Population density	Population divided by the subdistrict area
Diversity	Mixed land use	Mixed degree of POI within the 1-km buffer
Accessibility	Facility accessibility	Numbers of POI within the 1-km buffer
	Park accessibility	Numbers of parks within the 1-km buffer
	Public transit station accessibility	Numbers of public transit stations within the 1-km buffer
Distance to destination	Distance to the nearest park	–
	Distance to the nearest public transit station	–

#### Mediators: Perception of Community Cohesion and Perception of Community Safety

This study explored two biopsychosocial pathways through which the built environment of the community affected the mental health of the older adults: perception of community cohesion and perception of community safety.

##### Perception of Community Cohesion

Following previous studies on community social cohesion (63, 64), we measured the perception of community cohesion by asking each respondent on what level they agreed with the four items that “You know a lot of people in the community,” “You have a harmonious relationship in the community,” “You belong to this community,” and “You think the cohesion of this community is very strong.” The respondents were asked to respond to these questions on the Likert scale: strongly disagree = 1; disagree = 2; generally agree = 3; agree = 4; and strongly agree = 5. We treated this variable as a continuous variable, since it was basically normally distributed. Then, we chose the Likert scoring method to calculate the score of perception of community cohesion since it contained the maximum information for the linear regression model (Cronbach's alpha was 0.714 in this study, indicating good internal consistency). The Likert scoring method is used by adding items of the same construct (59, 65). The total score of the perception of community cohesion was generated by adding four items ranging from 4 to 20. Higher scores indicated a better perception of community cohesion.

##### Perception of Community Safety

We measured with the following questions: “You think the community environment is quiet” and “You think the community has good public security.” Based on the Likert scale, each question had five options, scored from 1 to 5: strongly disagree, disagree, generally agree, agree, and strongly agree. It also contained the maximum information for the linear regression model (Cronbach's alpha = 0.853). The total score of the perception of community safety was generated by adding two items ranging from 2 to 10. Higher scores indicated a better perception of community safety.

#### Moderator: Income

Previous studies indicated that the perception of community cohesion and safety might have different effects on the mental health of older adults depending on the level of individual income (3, 66). We therefore introduced income into the model as a moderating variable and interacted income with mediating variables to assess the moderating effect of income. We divided the income into four groups by the quartile division method, with the first group (the lowest income level) as the reference group. This was mainly based on two considerations. First, the relationship between the perception of community cohesion and safety and mental health might be non-linear. Second, the income might not be normally distributed.

#### Individual Covariates

The regression results were adjusted for the following individual covariates: age, sex, education level, marital status, hukou type, monthly income, and living style. Hukou type refers to two types of household: local and non-local hukou, which are separated from population migration and household registration migration. Living style refers to living alone or with spouse or children.

### Methods

We estimated the relationship between built environment and mental health using multilevel linear models. Multilevel models were more suitable than single-level models in this case due to the nested characteristics of the data. They can identify differences between groups (communities) or within groups (individuals) and can explain the multi-factor mechanism of health (67). Therefore, they are widely used in existing studies about residents' health (53, 58, 68). The full models were specified as follows:


$$\begin{array}{l} Y_{ij}=\beta_{0}+\beta_{1}W_{j}+\beta_{2}M_{ij}+\beta_{3}X_{ij}+\mu_{j}+\varepsilon_{ij} \end{array}$$

where *Y*_*ij*_ represents mental health of older adults *i* in community *j, W*_*j*_ represents the built environment of community *j, M*_*ij*_ represents the perception of community cohesion and safety of older adults *i* in community *j*, and *X*_*ij*_ represents socioeconomic attributes of older adults *i* in community *j*, β_0_ is the constant, β_1_, β_2_, and β_3_ represent the coefficients of the variables, respectively, and μ_*j*_and ε_*ij*_ represent the random effects at the community and individual levels, respectively.

We also used mediation analysis to test the mediating effect of mediators. Following the approach of Baron and Kenny (69), we used stepwise regression to test the mediation effect with Stata12.0. First, we regressed the mental health on the built environment and covariates (model 1). Second, we regressed two mediators on the built environment and covariates (model 2a−2b). Third, we regressed mental health on the built environment, two mediators, and covariates (model 3a−3b). We used the bootstrap method to test whether the effect of the mediating variable was significant. In addition, we added the interaction term between the income level and the mediating variables to model 3a−3b to obtain the model 4a−4b and used the Wald test to test the significance of the interaction term so as to test the moderating effect of the income level.

## Results

### Descriptive Statistics

Table 3 demonstrates the profile of the respondents. The respondents were dominated by older adults aged 60–75 years, accounting for about 75% of all respondents. The proportion of men and women was almost equal. The older adults with a local hukou accounted for about 70%, and the proportion of older adults with elementary school and below was the highest (41.416%), followed by those with a junior high school (28.433%); the lowest percentage was those with a college degree or above (2.682%). The average income of the respondents was about 3000 RMB. The older adults living with their children accounted for about 60% of all respondents, and the older adults living alone or with their spouses accounted for about 40%. The average score for the mental health of the older adults was 31.710, and their score of perception of community cohesion and safety was 15.578 and 7.739, respectively.

**Table 3:** Summary statistics for all variables.

**Variables**	**Mean (SD)/*N* (%)**
**Dependent variable**	
Mental health	31.710 (4.950)
**Independent variable**	
Population density	1.944 (1.824)
Mixed land use	0.667 (0.085)
Facility accessibility	4044.568 (3546.420)
Park accessibility	4.734 (4.519)
Public transit station accessibility	28.733 (16.137)
Distance to the nearest park	0.482 (0.578)
Distance to the nearest public transit station	0.267 (0.225)
**Mediating variable**	
Perception of community safety	7.739 (0.707)
Perception of community cohesion	15.578 (2.828)
**Individual variable**	
Age (year)	
60–75	703 (75.429%)
Above 75	229 (24.571%)
Sex	
Female	527 (56.545%)
Male	405 (43.455%)
Hukou type	
Non-local hukou	284 (30.472%)
Local hukou	648 (69.528%)
Marital status	
Unmarried, widowed, or divorced	215 (23.069%)
Married	717 (76.931%)
Educational level	
Primary school and below	386 (41.416%)
Junior middle school	265 (28.433%)
High school or technical secondary school	218 (23.391%)
Training school	38 (4.077%)
Bachelor's degree or above	25 (2.682%)
Monthly income	3183.5 (2568.919)
Living style	
Living alone or with a spouse	389 (41.738%)
Living with children	543 (58.262%)

**Figure 3:**
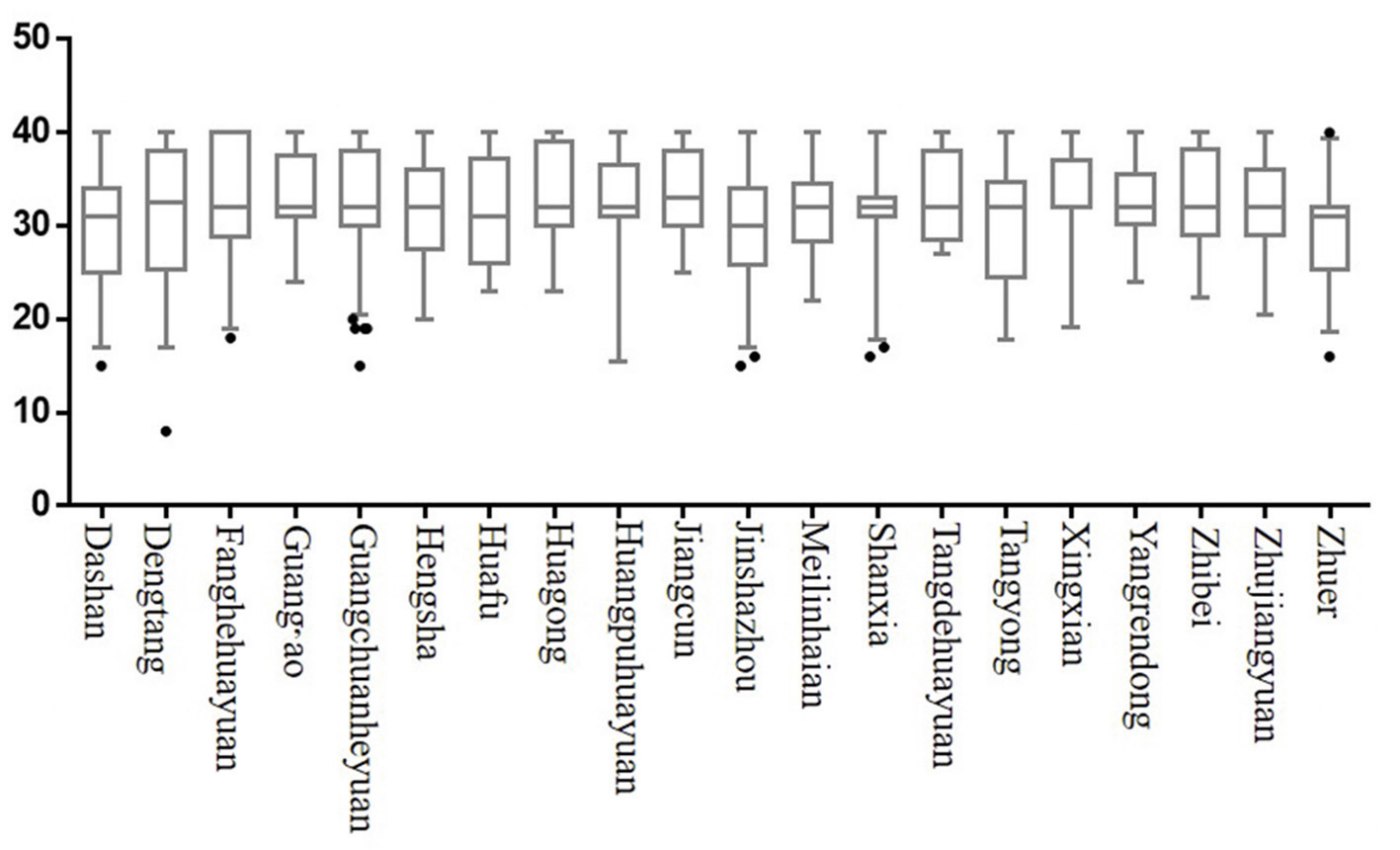
Level of mental health of the older adults in the sample communities.

The box diagram in Figure 3 shows the spatial differences in the mental health of the older adults in 20 sample communities in this survey. In the sample distribution, the highest score on mental health appeared in Zhuer village and the lowest in Dengtang village.

### Relationship Between Built Environment and Mental Health

We calculated the intra-class correlation coefficient (ICC) of the null model to determine whether multilevel models were necessary. The ICC for the null model (0.1196) indicated that the multilevel models were more suitable than the single-level models. Further, we explored the relationship between built environment and the mental health of older adults. Table 4 shows the results of the multilevel linear regression models. Model 1 was the benchmark model, which estimated the relationship between the built environment and the mental health of older adults after controlling the individual covariates. As for the built environment, facility accessibility (β = 0.005, *P* < 0.1) and distance to the nearest park (β = 1.389, *P* < 0.05) were positively correlated to the mental health of older adults. Public transit station accessibility (β = −0.086, *P* < 0.05) and distance to the nearest public transit station (β = −3.037, *P* < 0.1) were negatively correlated to the mental health of older adults. As for individual covariates, women were more likely to have better mental health than men. Those who held local hukou, higher education, and higher income were more likely to report better mental health than their counterparts.

**Table 4:** Relationship between built environment, perception of community cohesion, perception of community safety, and mental health.

**Variables**	**Model 1 (DV: Mental health)**		**Model 2a (DV: Perception of community cohesion)**		**Model 2b (DV: Perception of community safety)**	
	**Coef**.	**SE**	**Coef**.	**SE**	**Coef**.	**SE**
**Built environment**						
Population density	0.100	0.249	0.120	0.161	0.089	0.070
Mixed land use	5.333	4.423	−0.509	3.286	4.727***	1.243
Facility accessibility	0.005*	0.001	−0.001	0.000	0.0003***	0.00005
Park accessibility	−0.028	0.075	−0.014	0.055	−0.043**	0.021
Public transit station accessibility	−0.086**	0.033	−0.003	0.024	−0.408***	0.009
Distance to the nearest park	1.389**	0.636	0.568**	0.474	0.617**	0.179
Distance to the nearest public transit station	−3.037*	1.688	−2.712**	1.343	−0.888*	0.474
**Individual variable**						
Age (ref. 60–75)						
Above 75	0.237	0.472	−0.123	0.181	0.012	0.132
Sex (ref. female)						
Male	−0.638**	0.399	−0.515**	0.155	−0.128	0.112
Hukou (ref. non-local)						
Local hukou	0.184*	0.471	1.195***	0.194	−0.027	0.132
Marital status (ref. unmarried, widowed, or divorced)						
Married	0.354	0.457	0.079	0.175	0.014	0.128
Educational level (ref. primary school and below)						
Junior middle school	0.443	0.503	−0.317	0.193	−0.452**	0.141
High school or technical secondary school	0.565*	0.556	−0.180	0.215	−0.484**	0.156
Training school	−0.230	1.042	−0.617	0.401	−0.479	0.293
Bachelor's degree or above	1.250	1.280	−0.547	0.491	0.428	0.360
Monthly income	1.042***	0.225	0.394***	0.089	0.021*	0.063
Living style (ref. living alone or with a spouse)						
Living with children	0.207	0.408	−0.029	0.157	−0.148	0.115
Constant	20.380***	3.326	13.278***	2.398	5.266***	0.935
Log likelihood	−2958.3714		−2072.4105		−1776.6894	
Prob > chi2	0.0000		0.0000		0.0000	
AIC	5956.743		4184.821		3593.379	

***Means significant at the 1% threshold level; **means significant at the 5% threshold level; *means significant at the 10% threshold level.

### Mediating Effect of Perception of Community Cohesion and Safety

Model 2a−2b estimated the relationship between the built environment and two mediators (perception of community cohesion and perception of community safety).

As shown in Table 4, in model 2a, distance to the nearest park (β = 0.568, *P* < 0.05) was positively correlated to the perception of community cohesion. Distance to the nearest public transit station (β = −2.712, *P* < 0.05) was negatively correlated to the perception of community cohesion. Women (β = 0.515, *P* < 0.05), with local hukou (β = 1.195, *P* < 0.01) and more income (β = 0.394, *P* < 0.01), were more likely to perceive community cohesion. In model 2b, mixed land use (β = 4.727, *P* < 0.01), facility accessibility (β = 0.0003, *P* < 0.01), and distance to the nearest park (β = 0.617, *P* < 0.05) were positively correlated to perception of community safety. Park accessibility (β = −0.043, *P* < 0.05), public transit station accessibility (β = −0.408, *P* < 0.01), and distance to the nearest public transit station (β = −0.888, *P* < 0.1) were negatively correlated to perception of community safety. Those having higher income (β = 0.021, *P* < 0.1) perceived their community as safer. The older adults with high school or technical secondary school education perceived their community less safe.

### Relationship Between the Built Environment, Mediators, and Mental Health

Model 3a-3b estimated the relationship between the built environment and the mental health of older adults while taking into account two mediators: perception of community cohesion and safety (Table 5).

**Table 5:** Mediation effect of two mediators: perception of community cohesion and perception of community safety.

**Variables**	**Model 3a (Mediator: Perception of community cohesion)**		**Model 3b (Mediator: Perception of community safety)**	
	**Coef**.	**SE**	**Coef**.	**SE**
**Built environment**				
Population density	0.043	0.244	0.037	0.244
Mixed land use	5.456	4.339	1.995	4.368
Facility accessibility	0.0004**	0.0002	0.0002	0.0002
Park accessibility	−0.039	0.738	0.002	0.074
Public transit station accessibility	−0.084**	0.033	−0.058*	0.033
Distance to the nearest park	1.273**	0.624	0.553*	0.627
Distance to the nearest public transit station	−2.231	1.661	−2.410	1.657
**Perception of the built environment**				
Perception of community cohesion	0.499***	0.083		
Perception of community safety			0.706***	0.114
**Individual variable**				
Age (ref. 60–75)				
Above 75	0.290	0.463	0.228	0.463
Sex (ref. female)				
Male	−0.394	0.394	−0.547	0.391
Hukou (ref. non-local)				
Local hukou	−0.512	0.476	0.203	0.461
Marital status (ref. unmarried, widowed, or divorced)				
Married	0.338	0.448	0.344	0.447
Educational level (ref. primary school and below)				
Junior middle school	0.607	0.494	0.462	0.495
High school or technical secondary school	0.664	0.546	0.905*	0.548
Training school	0.179*	1.025	0.108	1.023
Bachelor's degree or above	1.614*	1.257	0.947	1.255
Monthly income	0.858***	0.223	1.057***	0.220
Living style (ref. living alone or with a spouse)				
Living with children	0.217**	0.400	0.312	0.400
Constant	13.879***	3.436	16.661***	3.315
Log likelihood	−2940.488		−2939.6657	
Intergroup variance	8.55e−09		0.0000753	
Intragroup variance	5.6943		5.689357	
Prob > chi2	0.0000		0.0000	
AIC	5922.976		5921.331	

***Means significant at the 1% threshold level; **means significant at the 5% threshold level; *means significant at the 10% threshold level.

In model 3a, facility accessibility (β = 0.0004, *P* < 0.05) and distance to the nearest park (β = 1.273, *P* < 0.05) positively correlated with mental health. Public transit station accessibility (β = −0.084, *P* < 0.05) negatively correlated with mental health. Perception of community cohesion (β = 0.499, *P* < 0.01) positively correlated with the mental health of older adults. The older adults who were highly educated (β = 0.179, *P* < 0.1; β = 1.614, *P* < 0.1) and richer (β = 0.858, *P* < 0.01) reported a higher level of mental health. Those who lived with their children (β = 0.217, *P* < 0.1) also reported better conditions of mental health.

In model 3b, public transit station accessibility (β = −0.058, *P* < 0.1) was negatively correlated to mental health, while distance to the nearest park (β = 0.553, *P* < 0.1) was positively correlated to mental health. Perception of community safety (β = 0.706, *P* < 0.01) was positively correlated to mental health. The older adults who were well educated (β = 0.905, *P* < 0.1) and had higher income (β = 1.057, *P* < 0.01) were more likely to report a high level of mental health. We estimated the mediation effect of the two mediators separately using the bootstrap method (Table 6), revealing that each of the two mediators played a mediating role.

**Table 6:** Results of the bootstrap test.

**95% Confidence Interval**	**Perception of community cohesion**	**Perception of community safety**
Facility accessibility	(−0.0000462, 0.000015)	(0.0000259, 0.0000116)
Public transit station accessibility	(−0.0045629, 0.0026365)	(0.0009572, 0.0022083)
Distance to the nearest park	(0.0466861, 0.0686167)	(−0.1286379, 0.0622343)
Distance to the nearest public transit station	(−0.7201084, 0.2243348)	(−0.1839447, 0.1587168)

### Moderating Effect of the Income Level

We added the interaction items of two mediators (perception of community cohesion and safety) and moderator (income) to the model 4a–4b (Table 7) to test whether the income level had a moderating effect on the relationship between the perception of community cohesion and safety and the mental health of older adults. The Wald test was used to test the significance of interaction terms. The results showed that the relationship between the perception of community cohesion and safety and the mental health of older adults varied with the income level. The Wald test indicated that all the interaction terms in the model 4a−4b passed the significance test.

**Table 7:** Relationship between the perception of community cohesion and safety and mental health: the moderating effect of income.

**Variable**	**Dependent variable: mental health**	
	**Coef**.	**SE**
**Model 4a**		
Perception of community cohesion	0.296**	0.143
Perception of community cohesion × income(ref:Q1)		
Perception of community cohesion × Q2	0.131	0.221
Perception of community cohesion × Q3	0.499**	0.214
Perception of community cohesion × Q4	0.351*	0.202
**Model 4b**		
Perception of community safety	1.052***	0.254
Perception of community safety × income(ref:Q1)		
Perception of community cohesion × Q2	−0.633*	0.334
Perception of community cohesion × Q3	−0.568*	0.333
Perception of community cohesion × Q4	−0.147	0.335
Wald test	18.24	

***Means significant at the 1% threshold level; **means significant at the 5% threshold level; *means significant at the 10% threshold level.

In model 4a, compared with the first group (lowest income level), the coefficient of the interaction of the third and fourth groups of income and the perception of community cohesion was significant. That is, only when it was higher than the average income level, the income level could moderate the relationship between the perception of community cohesion and mental health. The moderating effect was the strongest at the medium and high income levels.

In model 4b, compared with the first group (lowest income level), the coefficient of the interaction of the second and third groups of income and the perception of community safety was significant. That is, only when it was higher than the average income level, the income level could moderate the relationship between the perception of community safety and mental health. The moderating effect was the strongest at the average income level. In addition, with the increase in income, the moderating effect gradually weakened to an insignificant level.

## Discussion

Consistent with previous research findings from developed countries, our study also indicated a significant correlation between the built environment and the mental health of older adults in China (11, 70, 71). The research findings also suggested that both the perception of community cohesion and the perception of community safety had a significant mediating effect between the built environment and the mental health of older adults.

The research results indicated that facility accessibility and distance to parks significantly positively correlated with the mental health of older adults, which was consistent with the findings in the previous literature. With continued urbanization in China, the public facilities gradually improve with the increase in the size of cities. The high degree of facility accessibility around communities increases residents' proximity to recreational venues and public spaces and enhances the frequency of social interactions for older adults (72), which is conducive to improving their state of mental health (73). Distance to parks is an indicator of opportunities for older adults to enjoy green spaces. As important green spaces and public spaces, parks and squares have been shown to improve mental health (31, 74, 75). Parks and squares are important venues for social interactions in everyday life, which act as critical links to help residents maintain interpersonal relations and improve community cohesion, and therefore are conducive to improving the mental health of residents (76–78). The number of public transit stations and the distance to the stations significantly negatively correlated with the mental health of older adults, which was different from the results for developed countries. In megacities such as Guangzhou, the public transportation network shows continuous improvement with the increase in population density. However, if surrounded by too many public transit stations, communities may suffer from noise and exhaust pollution (79), decreasing the willingness of older adults to engage in social interactions, which is not conducive to improving their mental health (80–82). Long-term exposure to exhaust pollution is also detrimental to physical and mental health. Older adults are especially vulnerable to air pollution-related diseases (83–85) as well as depression and anxiety related to air pollution (86).

Our study verified the paths through which the perception of community cohesion and community safety mediated the relationship between the built environment and the mental health of older adults. In other words, creating a built environment that provides residents with opportunities to perceive a harmonious and safe community environment is an important path through which a built environment affects the mental health of residents. The perception of community cohesion has a partial mediating effect between the shortest distance to parks and mental health and many studies have revealed the potential reasons for this effect. For example, older adults are more likely to engage in social interactions in parks. These public spaces help them develop an emotional network among neighbors, ease their negative emotions, and enhance their positive mental state (87, 88). Also, older adults can obtain social support, reinforcing their attachment to the community and sense of belonging in these public spaces. As such, they have a stronger perception of community cohesion and maintain a positive emotional and mental state (58, 77). Interestingly, our research findings suggested that perceived community safety had a mediating effect between the built environment and mental health, which was not reported in previous empirical research in China (37). The relationships between accessibility to facilities, the number of public transit stations and mental health are mediated by the perception of community safety. A higher degree of accessibility to facilities improves the orderliness of areas surrounding a community and makes it more discernable and interesting. It helps keep “more eyes on the streets,” enhances residents' sense of community safety, and eases residents' psychological burden (89). In contrast, when the density of public transit stations exceeds a threshold, a crowding effect occurs (90). A high density of public transit stations and the resulting high population mobility create a noisy and unsafe community atmosphere. To a certain extent, as indicated by the broken windows theory, built environment that lacks order and social control creates distrust and fear among nearby residents (91) and creates pressure and mental stress on residents. This finding is different from the research conclusions for developed countries. The main reason for the difference is that urban residents, including older adults, regardless of their socioeconomic status, in China are likely to live in noisier and more crowded urban areas than residents in developed countries. Given this difference, a high density of public transit stations is not conducive to improving mental health (3).

We also identified the moderating effect of income. The enhancing effect of the perception of community cohesion on mental health was the strongest for older adults who had medium to high levels of income. Older adults with higher levels of income had more options with regard to adjusting their mental state (e.g., travel or recreational activity), reducing the chances of suffering from mental health problems (92, 93). The moderating effect of income was weaker for older adults with lower levels of income because these people, with economic and information accessibility limitations, tended to neglect mental health problems and were more likely to lack the resources needed to cope with these issues (94, 95). With an increase in income, the mediating effect of the perception of community safety that helped improve mental health decreased. Many research findings from developed countries also identified the mechanism underlying this phenomenon: people with a lower socioeconomic status suffered from the inferior built environment (crowdedness and noise) and were more likely to be subject to environmental stress, leading to a worse mental state (66).

This study had the following limitations. First, it was based on an analysis of cross-sectional data, making it difficult to draw a causal relationship between a built environment and the mental health of older adults. Second, the two mediating variables in this study (perception of community cohesion and perception of community safety) were indicators self-reported by the older adults. Hence, more objective measurements should be incorporated in future research. Third, although this study controlled for the socioeconomic characteristics of individuals and families to minimize the impact of self-selection, further experiments are needed to clarify the relationship between a built environment and mental health. Moreover, the research location selected for this case study was Guangzhou, one of the megacities of China. The research results need to be further verified for applicability to small- and medium-sized cities. Finally, the rapid and drastic changes in the built environment of Guangzhou have occurred under the stimulus of rapid urbanization and government policies. At the same time, in Chinese culture and consciousness, the adults still bear important responsibilities in the family. Therefore, considering the particularity of the development environment, government policies, history and culture, and the limited sample size of this study, the research conclusions cannot be extrapolated to other countries to a certain extent.

## Conclusions

This study employed a multilevel model and mediating effect analysis to examine the relationship between built environment and the mental health of older adults, as well as the mediating paths between the two. The results indicated a significant relationship between the built environment and the mental health of older adults. Also, the perception of community cohesion and community safety played a mediating role in this relationship. Furthermore, the results from the moderating effect analysis indicated that income moderated the relationship between the perceptions of community cohesion and safety and the mental health of older adults. The research results demonstrated that the built environment of a community played an important role in improving the mental health of older adults in China. We recommend that when governments develop public policies and when planners conduct urban planning, they should fully evaluate and balance the positive and negative effects brought about by infrastructure construction in dense cities. They should also make every effort to adapt to older adults' attachment to spaces and meet their need for social support so as to maximize the positive effect of the built environment on the mental health of older adults and hence effectively cultivate a healthy aging society. In addition, different from a high-density urban environment, a rural environment has the characteristics of low population density, road network, and infrastructure layout. Empirical research should be conducted in the future to compare the differences in the effects of the urban and rural environments on the health of older adults so as to propose strategies for constructing differentiated urban and rural health environment and provide a reference for the policy planning of relevant departments.

## Data Availability Statement

The data analyzed in this study is subject to the following licenses/restrictions. The datasets presented in this article are not readily available because of institutional copyright issues. Requests to access the datasets should be directed to CZ, zhoucs@mail.sysu.edu.cn.

## Author Contributions

RZ and CZ developed the main ideas of the study, gathered the data, performed the model construction and estimation, and wrote the manuscript. XH and YL helped to collect and process data. ML participated in revising the manuscript and proofreading the article. All authors have read and agreed to the published version of the manuscript.

## Funding

This work was supported by the National Social Science Foundation of China (17BRK010).

## Conflict of Interest

The authors declare that the research was conducted in the absence of any commercial or financial relationships that could be construed as a potential conflict of interest.

## Correction note

A correction has been made to this article. Details can be found at: 10.3389/fpubh.2026.1797057.

## Publisher's Note

All claims expressed in this article are solely those of the authors and do not necessarily represent those of their affiliated organizations, or those of the publisher, the editors and the reviewers. Any product that may be evaluated in this article, or claim that may be made by its manufacturer, is not guaranteed or endorsed by the publisher.
