# Correction: The relationship between built environment and mental health of older adults: mediating effects of perceptions of community cohesion and community safety and the moderating effect of income

**DOI:** 10.3389/fpubh.2026.1797057

**Published:** 2026-02-12

**Authors:** Rongrong Zhang, Xiong He, Ying Liu, Ming Li, Chunshan Zhou

**Affiliations:** 1 School of Geography and Planning, Sun Yat-sen University, Guangzhou, China; 2 Land Consolidation and Rehabilitation Center MNR, Beijing, China

**Keywords:** built environment, older adults, mental health, perception of community cohesion, perception of community safety, income

There was a mistake in the caption of Figure 2 as published. The attribution for the map used was missing. The corrected caption of Figure 2 appears below.

Location of surveyed communities. Map from the National Natural Resources and Geospatial Basic Information Database (https://www.sgic.net.cn/web/geo/index.html#/Home).

The original version of this article has been updated.

