# Supplementary material for: The Relationship Between Built Environment and Mental Health of Older Adults: Mediating Effects of Perceptions of Community Cohesion and Community Safety and the Moderating Effect of Income
Source: Front Public Health. 2022 Jun 17;10:881169. doi: 10.3389/fpubh.2022.881169 (PMC9247295; doi:10.3389/fpubh.2022.881169)
Supplement: Supplementary file 1 [file Data_Sheet_1.pdf]

Appendix A. Measurement Scales

Mental health:

1. Mental health

(i) Your mind has been in a healthy state in the past four weeks.

(1) Strongly disagree; (2) disagree; (3) Generally agree; (4) Agree; (5) Strongly agree.

(ii) You have felt calm in the past four weeks.

(1) Strongly disagree; (2) disagree; (3) Generally agree; (4) Agree; (5) Strongly agree.

(iii) You have felt happy in the past four weeks.

(1) Strongly disagree; (2) disagree; (3) Generally agree; (4) Agree; (5) Strongly agree.

(iv) You have been able to concentrate on your work in the past four weeks.

(1) Strongly disagree; (2) disagree; (3) Generally agree; (4) Agree; (5) Strongly agree.

(v) You haven't felt stressed in the past four weeks.

(1) Strongly disagree; (2) disagree; (3) Generally agree; (4) Agree; (5) Strongly agree.

(vi) You haven't felt nervous in the past four weeks.

(1) Strongly disagree; (2) disagree; (3) Generally agree; (4) Agree; (5) Strongly agree.

(vii) You haven't felt dejected in the past four weeks.

(1) Strongly disagree; (2) disagree; (3) Generally agree; (4) Agree; (5) Strongly agree.

2. Vitality

(viii) You haven't felt energetic in the past four weeks.

(1) Strongly disagree; (2) disagree; (3) Generally agree; (4) Agree; (5) Strongly agree.

Perception of community cohesion:

(i) You know a lot of people in the community.

(1) Strongly disagree; (2) disagree; (3) Generally agree; (4) Agree; (5) Strongly agree.

(ii) You have a harmonious relationship in the community.

(1) Strongly disagree; (2) disagree; (3) Generally agree; (4) Agree; (5) Strongly agree.

(iii) You belong to this community.

(1) Strongly disagree; (2) disagree; (3) Generally agree; (4) Agree; (5) Strongly agree.

(iv) You think the cohesion of this community is very strong.

(1) Strongly disagree; (2) disagree; (3) Generally agree; (4) Agree; (5) Strongly agree.

Perception of community safety:

(i) You think the community environment is quiet.

(1) Strongly disagree; (2) disagree; (3) Generally agree; (4) Agree; (5) Strongly agree.

(ii) You think the community has good public security.

(1) Strongly disagree; (2) disagree; (3) Generally agree; (4) Agree; (5) Strongly agree.
